# Herbal medicine for the management of postoperative pain: A protocol for the systematic review of randomized controlled trials

**DOI:** 10.1097/MD.0000000000014016

**Published:** 2019-01-04

**Authors:** Kyung Moo Park, Ji Hwan Kim

**Affiliations:** a Department of Korean Medicine Rehabilitation; b Department of Sasang Constitutional Medicine, College of Korean Medicine, Gachon University, Seongnam, Korea.

**Keywords:** herbal medicine, postoperative pain, protocol, systematic review

## Abstract

**Background:**

Postoperative pain is caused by surgical trauma and poses a significant challenge for health care providers. Opioid analgesics are commonly used to control postoperative pain. However, these drugs are associated with a number of negative side effects. This review will assess the clinical evidence for and against the use of herbal medicine formulas as a treatment for postoperative pain, especially after musculoskeletal surgery.

**Methods:**

Twelve databases will be searched from inception to March 2019. We will include randomized controlled trials (RCTs) assessing herbal medicine decoctions used to treat any type of postoperative pain. All RCTs of decoctions or modified decoctions will be included. The methodologic qualities of the RCTs will be assessed using the Cochrane Collaboration tool for assessing risk of bias, while confidence in the cumulative evidence will be evaluated using the Grading of Recommendations Assessment, Development, and Evaluation method.

**Conclusion:**

This systematic review will be published in a peer-reviewed journal and disseminated electronically and in print. The review will be updated to inform and guide health care practices.

Registration number: CRD42018094897

## Abstract 2

Supplemental Digital Content is available in the text

## 1 Introduction

During surgery, inflammatory mediators such as histamine, leukotriene, prostaglandin, cytokine, and bradykinin are released from damaged tissue.^[1]^ These released inflammatory mediators activate the peripheral nociceptors, which leads to hyperalgesia.^[1,2]^ In the afferent nerve, glutamate, aspartate, and substance P are released, and the pain stimulus is transmitted and modulated.^[3,4]^ Nociceptive pain is recognized in the higher centers of the brain and is modulated by endogenous opioids, noradrenaline, serotonin, and the like.^[5,6]^ Postoperative pain also occurs through a variety of complex pathways.

Inadequate postoperative pain control can result in decreased immunity, retention of sodium and water, delayed wound recovery, gastrointestinal obstruction, cardiovascular anomalies due to adrenergic activity, and pulmonary complications due to increased functional residue and respiratory secretion.^[7,8]^ In addition to opioid agonists, nonsteroidal anti-inflammatory drugs, and acetaminophen, medications such as ketamine, clonidine, lidocaine, and gabapentinoids may be used as adjuvants for postoperative pain management.^[9,10]^

According to the Korea National Statistical Office, the number of spinal operations per 100,000 people increased by 181% from 2006 to 2016, total hip arthroplasty increased by 408%, and total-knee arthroplasty increased by 228%.^[11,12]^ As the rate of musculoskeletal surgery increased, the 5-year reoperation rates for patients with a herniated disk and spinal stenosis increased to 13.7% and 14.2%, respectively.^[13,14]^ Since the number of surgical patients due to musculoskeletal injuries is increasing, there is a greater need for correct rehabilitation and postoperative care.^[15]^ There are some reported cases of postoperative pain therapy and rehabilitation in Korean medicine, but there is a lack of systematically organized domestic studies.^[16]^ Therefore, the authors aim to perform a systematic review of studies examining the effects of herbal medicine treatment on postoperative pain.

## 2 Methods

### 2.1 Study registration

The current protocol report adheres to the PRISMA protocols.^[17]^ The protocol for this systematic review has been registered on PROSPERO, the international prospective register of systematic reviews, under the number CRD42018094897.

### 2.2 Dissemination and ethical approval

This systematic review will be published in a peer-reviewed journal and disseminated electronically and in print. The review will be updated to inform and guide health care practices. As this is a study based on the review of published literature, ethical approval is not required.

### 2.3 Data sources

The following databases will be searched from inception to the present date: MEDLINE, Embase, and the Cochrane Central Register of Controlled Trials (CENTRAL). We will also search 6 Korean medical databases (OASIS, the Korean Traditional Knowledge Portal, the Korean Studies Information Service System, KoreaMed, the Korean Medical Database, and DBPIA) and 3 Chinese databases, including CNKI (the China Academic Journal, the China Doctoral Dissertations and Master's Theses Full-text Database, the China Proceedings of Conference Full-Text Database, and the Century Journal Project), Wanfang and VIP. In addition, we will search a Japanese database and conduct nonelectronic searches of conference proceedings. The search strategy that will be applied in the MEDLINE database is presented in Supplement 1. Similar search strategies will be used in the other databases.

### 2.4 Types of studies

Prospective randomized controlled trials (RCTs) that evaluate the effectiveness of herbal medicine formulas as a treatment for postoperative pain, especially after musculoskeletal surgery, will be included in this review. Both treatment with herbal medicine alone and concurrent treatment with other therapy will be considered acceptable if only herbal medicine is applied to the intervention group and any other treatment is provided equally to both intervention and control groups. Trials with any type of control intervention will be included. No language restrictions will be imposed.

### 2.5 Types of participants

The study will include all participants who have acute/subacute pain (<3 months) or chronic pain (more than 3 months) undergoing any type of surgery (e.g., back surgery, ambulatory knee surgery, hip arthroplasty, foot and ankle surgery, lumbar spinal fusion, low back intervertebral disc surgery, shoulder surgery, oral/maxillofacial surgery, etc).

### 2.6 Types of interventions

The RCTs that include herbal medicine as the sole treatment or as an adjunct to other treatments will be included, as long as the RCTs including other treatments provide the same treatment to the control and intervention groups. Trials comparing herbal medicine with any type of control intervention will also be included. Studies that evaluate any type of formulation (i.e., decoction, tablet, pill, or powder) of herbal medicine will be eligible for inclusion.

### 2.7 Data extraction and quality assessment

Hard copies of all articles will be obtained and read in full. Two authors (KMP and JHK) will perform the data extraction and quality assessment using a predefined data extraction form. In addition, all interventions applying acupuncture will be extracted using the Standards for Reporting Interventions in Clinical Trials of Acupuncture guidelines. Risk of bias will be assessed using the Cochrane Handbook risk of bias assessment tool version 5.1.0, which takes into account random sequence generation, allocation concealment, blinding of participants and personnel, blinding of outcome assessment, incomplete outcome data, selective reporting, and other sources of bias.^[18]^ The results of the assessments will be presented using the scores of “L” indicating a low risk of bias, “U” indicating an uncertain risk of bias, and “H” indicating a high risk of bias. Disagreements will be resolved via discussion among all authors.

### 2.8 Data collection and synthesis

#### 2.8.1 Outcome measures

##### 2.8.1.1 Primary outcomes

We will measure the severity of postoperative pain using any valid scales such as the visual analog scale (0–100 mm or 0–10 cm), the Numeric Pain Rating Scale, or drug consumption (e.g., anesthetic demands for opioids, morphine, or other pain control drugs).

##### 2.8.1.2 Secondary outcomes

1. Quality of life
2. Patient-reported outcome measures
3. Adverse events

#### 2.8.2 Data synthesis

Differences between the intervention and control groups will be assessed. Mean differences (MDs) with 95% confidence intervals (CIs) will be used to measure the effects of treatment for continuous data. We will convert other forms of data into MDs. For outcome variables on different scales, we will use standard MDs with 95% CIs. For dichotomous data, we will present treatment effects as relative risks (RRs) with 95% CIs; other binary data will be converted into RR values.

All statistical analyses will be conducted using Cochrane Collaboration's software program Review Manager version 5.3 (Copenhagen, The Nordic Cochrane Centre, the Cochrane Collaboration, 2014) for Windows. We will contact the corresponding authors of studies with missing information to acquire and verify the data whenever possible. When appropriate, we will pool the data across studies to conduct a meta-analysis using fixed or random effects. We will use GRADEpro software from Cochrane Systematic Reviews to create a Summary of Findings table.

#### 2.8.3 Unit of analysis issues

For crossover trials, data from the first treatment period will be used. For trials that assessed more than 1 control group, the primary analysis will combine data from each control group. Subgroup analyses of the control groups will be performed. Each patient will be counted only once in the analyses.

#### 2.8.4 Addressing missing data

Intention-to-treat analyses including all randomized patients will be performed. For patients with missing outcome data, last observation carry-forward analysis will be performed. When individual patient data are initially unavailable, we will review the original source or the published trial reports for these data.

#### 2.8.5 Assessment of heterogeneity

Based on the data analysis, we will use random- or fixed-effect models to conduct the meta-analysis. Chi-squared and *I*-squared tests will be used to evaluate the heterogeneity of the included studies. *I*^2^ values > 50 will indicate high heterogeneity. When heterogeneity is observed, subgroup analyses will be conducted to explore the possible causes.^[19]^

#### 2.8.6 Assessment of reporting biases

Funnel plots will be generated to detect reporting biases when a sufficient number of included studies (at least 10 trials) is available.^[20]^ However, as funnel plot asymmetries are not equivalent to publication biases, we will aim to determine the possible reasons for any asymmetries in the included studies, such as small-study effects, poor methodological quality, and true heterogeneity.^[20,21]^

## 3 Discussion

In traditional East Asian countries such as Korea, China, and Japan, herbal medicine, acupuncture, moxibustion, and cupping therapy are often used for postoperative pain control and functional recovery.

In particular, acupuncture^[22,23]^ and electro acupuncture^[24]^ have been reported to have an analgesic effect for postoperative pain. Based on this, studies have examined electro acupuncture for pain patients after lumbar surgery.^[25,26]^

There are various existing studies on the mechanism and therapeutic effects of acupuncture for pain control, and previous studies on herbal medicines have examined the interaction of single herbal medicines in patients after surgery.^[27,28]^ However, there is a lack of research on the therapeutic effects of herbal medicine.^[29]^

Therefore, the authors would like to perform a systematic review regarding the effects of herbal medicine treatment on postoperative pain.

This evidence will be useful to patients, practitioners, and health policy makers.

Patients complaining of postoperative pain can receive appropriate herbal medicine treatment, and practitioner can confirm the basis of decision in treatment.

From a policy viewpoint, it can be used as evidence to establish an East-West integrative care model of postoperative pain.

The limitations of this systematic review are regional differences in the study of herbal medicine prescriptions in Eastern and Western countries.

## Author contributions

KMP, JHK conceived the study, developed the criteria, searched the literature, analyzed the data, and wrote the protocol. KMP and JHK conducted the preliminary search. KMP assisted in searching the Chinese literature and extracting the data. KMP and JHK revised the manuscript. All authors have read and approved the final manuscript.

**Conceptualization:** Ji Hwan Kim.

**Funding acquisition:** Kyung Moo Park.

**Investigation:** Ji Hwan Kim.

**Writing – original draft:** Ji Hwan Kim, Kyung Moo Park.

Ji Hwan Kim orcid: 0000-0001-7270-0987.

## Supplementary Material

Supplemental Digital Content
