# Supplementary material for: Herbal medicine for the management of postoperative pain: A protocol for the systematic review of randomized controlled trials
Source: Medicine (Baltimore). 2019 Jan 4;98(1):e14016. doi: 10.1097/MD.0000000000014016 (PMC6344157; doi:10.1097/MD.0000000000014016)
Supplement: Supplemental Digital Content [file medi-98-e14016-s001.docx]

**Supplement 1. Search strategy for the MEDLINE database**

#1 Search "Randomized controlled trial"

#2 Search "controlled trial"

#3 Search "randomised" OR "randomized"

#4 Search "randomly"

#5 Search "trial"

#6 Search (#1 OR #2 OR #3 OR #4 OR #5 OR #6)

#7 Search "postoperative"[Title/Abstract]

#8 Search "postoperative pain"[Mesh]

#9 Search (' pain, postoperative'[Title/Abstract] OR ‘postoperative pain’[Title/Abstract])

#10 Search (‘persistent postsurgical pain'[Title/Abstract] OR 'PPSP'[Title/Abstract])

#11 Search (#7 OR #8 OR #9 OR #10)

#12 Search "Medicine, Chinese Traditional"[Mesh]

#13 Search "Kampo medicine"

#14Search "Korean medicine" OR "Traditional Korean medicine"

#15 Search (#12 OR #13 OR #14)

#16 Search "Herbal Medicine"[Title/Abstract]

#17 Search "herbal*"[tw]

#18 Search "Herbal Medicine"[Mesh]

#19 Search (#16 OR #17 OR #18)

#20 #15 OR #19

#21 #6 AND #11 AND #20
